# The reperfusion rates after recombinant human tissue plasminogen activator thrombolysis depend not on a few but on a plethora of influencing factors

**DOI:** 10.1590/1806-9282.20240103

**Published:** 2024-05-20

**Authors:** Josef Finsterer, Fulvio Alexandre Scorza, Carla Alexandra Scorza

**Affiliations:** 1 Neurology and Neurophysiology Center, Department of Neurology – Vienna, Austria.; 2 Universidade Federal de São Paulo/Escola Paulista de Medicina, Neuroscience Discipline – São Paulo (SP), Brazil.

Dear Editor,

We read with interest Oliveira et al.'s article, which is a retrospective, cross-sectional, observational study on possible determinants of reperfusion after venous thrombolysis with recombinant human tissue plasminogen activator (rt-PA) based on a review of hospital records of inpatients diagnosed with ischemic stroke^1^. Analysis of 316 patient records revealed that reperfusion following rt-PA treatment occurred more frequently in women than men^1^. The mean admission severity score was higher in patients without reperfusion than in those with reperfusion. Mean ejection fraction (EF) was normal in reperfused and non-reperfused patients, but it was higher in non-reperfused compared to reperfused patients^1^. Reperfusion was associated with reduced mortality after ischemic stroke^1^. The study is impressive, but several points require discussion.

The main drawback of the study is its retrospective design. Retrospective design has several disadvantages. It does not allow control of the accuracy of the data stored, does not systematically apply the same examinations to all included patients, produces missing data, does not allow for the addition of missing data, and is not suitable for generating desirable new data.

The second limitation is that several factors that may additionally determine reperfusion rates were not included in the analysis^1^. Factors determining the reperfusion rates not included in the analysis were the degree of atherosclerosis, the number of risk factors for atherosclerosis, sympathetic activity, door-to-needle time, the extent of mismatch between stroke core and penumbra, which was confirmed by diffusion-weighted imaging (DWI) and perfusion-weighted imaging (PWI), stroke volume, blood pressure on admission, and rt-PA doses used.

The third limitation is that the modified thrombolysis in cerebral infarction (TICI) scoring system has not been used to assess the degree of reperfusion^2^. According to the modified TICI system, TICI3 means complete reperfusion, TICI2c means almost complete reperfusion except for slow flow in distal branches, TICI2b means partial filling covering >50% of the territory, TICI2a means partial filling of <50% of the territory, and TICI1 means no or minimal reperfusion^2^. The clinical outcome after thrombolysis can strongly depend on the TICI score achieved^3^.

A significant risk factor not included in the analysis was atrial fibrillation. Permanent atrial fibrillation can be a strong risk factor for re-occlusion and hence the outcome of thrombolysis. Therefore, it is mandatory to report the number of patients who had paroxysmal, persisting (>7 days response to treatment), or had permanent atrial fibrillation (not amenable to treatment).

Another factor affecting the reperfusion rate after thrombolysis is the presence or absence of pre-stroke antithrombotic or anticoagulant treatment. Reperfusion rates may depend largely on the coagulation status at the time of thrombolysis. Therefore, current medication must be included in the analysis as well as the number of the included patients who had hereditary or acquired coagulopathy.

There is a discrepancy in the number of patients included. In the first paragraph of the results, 316 patients were included (540 included and 224 excluded). In the next sentence, 192 were reperfused, 124 were not reperfused, and 12 did not show data consistent with reperfusion, yielding 328 patients. This inconsistency should be resolved. Why were the 12 patients with data inconsistent with reperfusion not combined with the 124 patients without reperfusion?

To sum up, the excellent study has limitations that should be addressed before final conclusions are drawn. Clarifying the weaknesses will strengthen the conclusions and improve the study. Reperfusion after systemic thrombolysis may depend not only on gender, age, and classic cardiovascular risk factors but also on a number of other influencing factors that need to be included in the analysis before drawing final conclusions.
